# Antileishmanial Activity of the Estrogen Receptor Modulator Raloxifene

**DOI:** 10.1371/journal.pntd.0002842

**Published:** 2014-05-08

**Authors:** Juliana Q. Reimão, Danilo C. Miguel, Noemi N. Taniwaki, Cristiana T. Trinconi, Jenicer K. U. Yokoyama-Yasunaka, Silvia R. B. Uliana

**Affiliations:** 1 Departamento de Parasitologia, Instituto de Ciências Biomédicas, Universidade de São Paulo, São Paulo, Brazil; 2 Núcleo de Microscopia Eletrônica, Instituto Adolfo Lutz, São Paulo, Brazil; Northeastern University, United States of America

## Abstract

**Background:**

The treatment of leishmaniasis relies mostly on parenteral drugs with potentially serious adverse effects. Additionally, parasite resistance in the treatment of leishmaniasis has been demonstrated for the majority of drugs available, making the search for more effective and less toxic drugs and treatment regimens a priority for the control of leishmaniasis. The aims of this study were to evaluate the antileishmanial activity of raloxifene *in vitro* and *in vivo* and to investigate its mechanism of action against *Leishmania amazonensis*.

**Methodology/Principal Findings:**

Raloxifene was shown to possess antileishmanial activity *in vitro* against several species with EC_50_ values ranging from 30.2 to 38.0 µM against promastigotes and from 8.8 to 16.2 µM against intracellular amastigotes. Raloxifene's mechanism of action was investigated through transmission electron microscopy and labeling with propidium iodide, DiSBAC_2_(3), rhodamine 123 and monodansylcadaverine. Microscopic examinations showed that raloxifene treated parasites displayed autophagosomes and mitochondrial damage while the plasma membrane remained continuous. Nonetheless, plasma membrane potential was rapidly altered upon raloxifene treatment with initial hyperpolarization followed by depolarization. Loss of mitochondrial membrane potential was also verified. Treatment of *L. amazonensis* – infected BALB/c mice with raloxifene led to significant decrease in lesion size and parasite burden.

**Conclusions/Significance:**

The results of this work extend the investigation of selective estrogen receptor modulators as potential candidates for leishmaniasis treatment. The antileishmanial activity of raloxifene was demonstrated *in vitro* and *in vivo*. Raloxifene produces functional disorder on the plasma membrane of *L. amazonensis* promastigotes and leads to functional and morphological disruption of mitochondria, which culminate in cell death.

## Author Summary

Cutaneous and visceral leishmaniasis are part of the group we call neglected diseases. They are serious conditions that afflict millions in vast regions of the world. These diseases are very difficult to treat. This is due to the scanty choice of effective drugs together with their potentially severe side effects. One way of finding new treatments for these neglected conditions is to repurpose drugs that are already in use to treat other diseases. In this paper, we show that raloxifene, a drug that is used for the treatment of osteoporosis and also as an alternative in the treatment of breast cancer, is active against the causative agents of leishmaniasis and is effective in the treatment of cutaneous leishmaniasis in an experimental model. We also show that the antileishmanial mechanism of action of raloxifene is related to damage to the cell membrane and to the mitochondrion of the parasite.

## Introduction

With an incidence of two million new cases per year, leishmaniasis is endemic in 98 countries and territories, representing an area where more than 350 million people are at risk of acquiring the infection [1]. The treatment of leishmaniasis relies mostly on parenteral drugs and involves high costs. Antimonial compounds were discovered nearly 100 years ago and remain the drug of choice for the treatment of leishmaniasis in many parts of the world, despite their high toxicity and consequent severe adverse effects. Amphotericin B and pentamidine are used as second-line drugs, but their administration also involves serious adverse effects [2]. Miltefosine has been approved for the therapy of visceral leishmaniasis in India, but its efficacy for the treatment of American leishmaniasis has been shown to be variable depending on the causative species [3]. Moreover, parasite resistance in the treatment of leishmaniasis was demonstrated for the majority of drugs available [4]. Therefore, the search for more effective and less toxic drugs and treatment regimens is a priority for the control of leishmaniasis [5].

Screening of drugs originally developed for another purpose, or drug repositioning, constitutes a promising strategy for the discovery of new compounds effective against neglected diseases. In the treatment of leishmaniasis, examples of this strategy are miltefosine, initially developed for the treatment of breast cancer, pentamidine, a hypoglycemic agent, as well as amphotericin B, used in the treatment of fungal infections [6]. The benefits of repositioning include the availability of materials and data such as toxicology studies, resulting in reduction of time and costs to bring the drug to the market [7].

Selective Estrogen Receptor Modulators (SERMs) are a class of therapeutic agents widely prescribed for the treatment and prevention of breast cancer, osteoporosis, and postmenopausal symptoms [8]. The most widely used SERM is tamoxifen, a triphenylethylene used in the management of breast cancer. Due to its mixed antagonist and agonistic estrogenic activity, long term use of tamoxifen has been associated with an increased risk of endometrial cancer in postmenopausal patients [9]. Drugs belonging to a second class of SERMs built on a benzothiophene scaffold are also in clinical use. One example of this second class is raloxifene, an oral SERM which has estrogen agonist effects on bone and cholesterol metabolism but behaves as a complete estrogen antagonist on mammary gland and uterine tissue [10], which results in skeletal benefit, with little, if any, uterine stimulation [11].

Previous studies have shown that tamoxifen is active against different species of *Leishmania in vitro* and *in vivo* [12], [13], [14]. In infections caused by *Leishmania amazonensis* in BALB/c mice, treatment with tamoxifen resulted in significant and sustained improvement in both clinical and parasitological parameters [15]. The activity of this drug has also been demonstrated in experimental infections by *Leishmania braziliensis*, the main causative agent of cutaneous leishmaniasis in Brazil. In this model, tamoxifen was able to significantly reduce the size of lesions and parasite burden [13]. Tamoxifen was also effective in the treatment of infections caused by *Leishmania major* in a murine model and in the treatment of visceral leishmaniasis in hamsters infected with *Leishmania infantum chagasi* [13], [14].

The activity of tamoxifen in the treatment of experimental leishmaniasis led us to investigate whether raloxifene also presents leishmanicidal effect. Here, we describe the antileishmanial activity of raloxifene *in vitro* and *in vivo* and investigate its mechanism of action against *L. amazonensis*, which is responsible for most cases of human cutaneous leishmaniasis in the Amazon region of Brazil [16].

## Materials and Methods

### Ethics statement

Animal experiments were approved by the Ethics Committee for Animal Experimentation (Protocol 033/42/02) of the Biomedical Sciences Institute of the University of São Paulo. The research adhered to the Brazilian Guidelines for Care and Utilization of Animals from the Conselho Nacional de Controle e Experimentação Animal (CONCEA).

### Drugs

Raloxifene hydrochloride and miltefosine were purchased from Sigma-Aldrich (St Louis, MO, USA). Stock solutions of raloxifene (10 mM) and miltefosine (20 mM) were prepared in DMSO and in sterile water, respectively, and kept at −20°C. Dilutions from the stock solutions were done in culture media. For *in vivo* experiments, fresh solutions of raloxifene were prepared in saline or Cremophor A25 (Sigma-Aldrich).

### Parasites and macrophages

Promastigotes of *Leishmania (Leishmania) amazonensis* (MHOM/BR/1973/M2269), *Leishmania (Leishmania) donovani* (LD-15/MHOM/SD/00), *Leishmania (Leishmania) infantum chagasi* (MHOM/BR/1972/LD), *Leishmania (Leishmania) major* (MHOM/IL/1981/Friedlin), *Leishmania (Leishmania) mexicana* (MHOM/BR/1974/M2682) and *Leishmania (Viannia) braziliensis* (MHOM/BR/1975/M2903) were maintained in M199 medium (Sigma-Aldrich) supplemented with 10% heat-inactivated fetal calf serum (FCS) (Invitrogen Corporation, NY, USA) and 0.25% hemin at 25°C. *L. braziliensis* and *L. infantum chagasi* cultures were also supplemented with 2% sterile male human urine. Promastigotes of a *L. amazonensis* transgenic line expressing luciferase (LaLUC) were grown in the same medium supplemented with 32 µg/mL G418 [17].

Amastigotes were purified from lesions as described [18]. Briefly, amastigotes were obtained from lesions induced in BALB/c mice, about 8 to 12 weeks after intradermal inoculation of 1×10^6^ parasites in the hind footpads. Lesions were removed and homogenized in phosphate-buffered saline (PBS); the suspension was cleared of cell debris by centrifugation at 50 *g* for 8 min; the supernatant was then washed three times in PBS and passed through a 25-gauge needle. Amastigotes recovered from tissue were resuspended in RPMI-1640 medium supplemented with 10% FCS, 2 mM glutamine and 50 mg/mL gentamicin and kept at 33°C in a 5% CO_2_ atmosphere.

Bone marrow-derived macrophages (BMDM) were obtained from BALB/c mice as previously described [19]. J774 macrophages were maintained in RPMI-1640 medium supplemented with 10% FCS at 37°C in a 5% CO_2_-humidified incubator.

### Evaluation of *in vitro* antileishmanial activity and mammalian toxicity

Cell viability was evaluated *in vitro* by cultivating promastigotes (5×10^6^ per well) or lesion-derived amastigotes (1×10^7^ per well) in M199 or RPMI-1640 medium, respectively, supplemented with 10% FCS. Parasites were incubated in the presence of increasing concentrations of raloxifene (7.75 to 62.0 µM for promastigotes and 3.75 to 30.0 µM for intracellular amastigotes, assayed at a 1.5-fold dilution) for 24 h. Miltefosine (assayed in concentrations varying from 1.5 to 45.0 µM) was used as a control drug. Quantification of viable cells was assessed either by cell counting or by measuring the cleavage of 3-(4,5-dimethylthiazol-2-yl)-2,5-diphenyl tetrazolium bromide (MTT; Sigma-Aldrich) as previously described [20]. MTT cleavage was assessed in a microplate reader (POLARstar Omega, BMG Labtech, Ortenberg, Germany) with a reference wavelength of 690 nm and a test wavelength of 595 nm.

The activity of raloxifene was also evaluated against *L. amazonensis* promastigotes (3×10^7^/mL) incubated in M199 medium or in Hank's balanced salt solution supplemented with 10 mM D-glucose (HBSS+Glc) (137 mM NaCl, 5.3 mM KCl, 0.4 mM KH_2_PO_4_, 4.2 mM NaHCO_3_, 0.4 mM Na_2_HPO_4_, pH 7.2, 10 mM D-glucose) for 2 hours. Quantification of viable cells was assessed by measuring the cleavage of (3-(4,5-dimethylthiazol-2-yl)-5-(3-carboxymethoxyphenyl)-2-(4-sulfophenyl)-2H-tetrazolium) with the CellTiter 96 Aqueous One Solution Cell Proliferation Assay (MTS, Promega), according to the manufacturer's instructions.

The half maximal effective concentrations (EC_50_) were determined from sigmoidal regression of the concentration-response curves using GraphPad Prism 5.0 software. Assays were performed in triplicate and results are expressed as the mean and standard deviation (SD) of at least three independent experiments.

*In vitro* cytotoxicity was evaluated by cultivating J774 macrophages (5×10^5^ per well) in 24-well plates for 24 h in the presence of increasing concentrations of raloxifene. Cell viability was assessed by the MTT assay as described above and results are expressed as percentage reduction in cell viability compared with untreated control cultures. The half maximal cytotoxic concentration (CC_50_) was determined as described above for EC_50_ values. The Selectivity Index of raloxifene was calculated as the ratio between the CC_50_ for J774 macrophages and the EC_50_ against *Leishmania* intracellular amastigotes.

Killing of intracellular *L. amazonensis* amastigotes was assayed by analysis of parasite burden in BMDM monolayers. Macrophages were plated on 96-well plates (8×10^4^ per well) in RPMI-1640 medium supplemented with 10% FCS and allowed to adhere overnight at 37°C, at 5% CO_2_. LaLUC stationary-phase promastigotes (in a ratio of 20 parasites: 1 macrophage) were added to the wells and the cultures were incubated at 33°C in a 5% CO_2_ atmosphere. After 3 h, parasites were removed by washing with RPMI-1640 medium and infected cultures were treated with increasing concentrations of raloxifene or miltefosine for 48 h. The monolayers were washed with PBS and luciferase detection was performed with the One Glo Luciferase Assay System (Promega Corporation), according to the manufacturer's instructions. Briefly, 20 µL of reagent at room temperature were added to each well containing 100 µL of PBS followed by homogenization. Luminescence units were determined immediately after adding the substrate in a Polarstar Omega reader (BMG Labtech).

Drug activity against *L. infantum chagasi* intracellular amastigotes was determined in BMDM infected macrophages in 16-well chamber Slides (NUNC) (8×10^4^ per well) and infection was carried out at 37°C as described above for *L. amazonensis*. For the evaluation of parasite burden under light microscopy, 16-well chamber slides were fixed in methanol and stained with the Instant Prov kit (Newprov, Pinhais-Paraná, Brazil). The percentage of infected macrophages was determined by counting 200 cells in each of the replicates.

### Transmission electron microscopy

Logarithmic-phase *L. amazonensis* promastigotes (2×10^7^/mL) were incubated with 60 µM raloxifene for different periods of time (30 min, 2 h and 14 h) at 25°C in M199 medium, supplemented with 10% FCS, in 24-well plates. Subsequently, promastigotes were centrifuged at 230 *g* for 10 min and the pellet was fixed in 2.5% glutaraldehyde : 4% paraformaldehyde in 0.1 M sodium cacodylate buffer (pH 7.4), rinsed in the same buffer and post-fixed in 1% osmium tetroxide, dehydrated in acetone series, and embedded in Epon resin.

BMDM (4×10^5^ cells per well) were seeded in 24-well microplates on ACLAR film (Electron Microscopy Sciences, USA) cut into discs and allowed to adhere overnight at 37°C at 5% CO_2_. Infection with *L. amazonensis* promastigotes was carried out at a ratio of 20∶1 parasites per macrophage. Infected macrophage cultures were kept at 33°C, 5% CO_2_ for 3 h in RPMI 1640 medium with 10% FCS and then washed twice with sterile PBS to remove free promastigotes. Infected cultures were treated with 9 µM raloxifene for 48 hours. The medium was removed and the monolayers were fixed as described above for promastigotes.

Ultrathin sections were obtained in a Sorvall Ultramicrotome, stained with uranyl acetate and lead citrate and observed under a JEOL transmission electron microscope operating at 80 kV. Images were recorded with a Gatan 785 ES1000W Erlangshen camera. Parasites or infected macrophages without treatment were used as a control.

### Identification of autophagic vacuoles

Monodansylcadaverine (MDC) staining was used to label autophagic vacuoles as previously described [21], [22]. *L. amazonensis* promastigotes (2×10^7^/mL) from early log phase cultures were treated with 30 and 60 µM raloxifene for 30 min in M199 medium supplemented with 10% FCS, at 25°C. As a positive control, parasites were subjected to starvation in PBS for 3 h [23]. Untreated parasites incubated in M199 medium for the same period were used as a negative control. Following the incubation period, cells were loaded with 100 µM MDC for 2 h. Cells were washed four times in PBS and fixed in 4% paraformaldehyde. One drop of fixed cells was added onto glass microscopic slides, covered by a coverslip and immediately visualized in a Zeiss AxioObserver microscope (Oberkochen, Germany) with excitation and emission wavelengths of 358 and 463 nm respectively and photographed using a digital camera (AxionCam HRc, Zeiss). Twenty fields were randomly chosen from each sample (magnification 630× or 1,000×). Assays were performed in two independent experiments.

### Evaluation of plasma membrane integrity

*L. amazonensis* promastigotes (5×10^6^/mL) were treated with 30 and 60 µM raloxifene for 20 min or 2 h in M199 medium, supplemented with 10% FCS at 25°C. Parasites treated with 25 µM digitonin were used as a positive control. Untreated parasites and parasites incubated with the highest volume of diluent (DMSO 0.6%) were used as negative controls. Parasites were stained with 10 µM propidium iodide (PI) and immediately analysed by flow cytometry using a Guava EasyCyte Mini Flow Cytometer System (Millipore). A total of 5,000 events were acquired in the region previously established as corresponding to the parasites. Fluorescence was quantified using the CytoSoft 4.2.1 software (Guava Technologies Inc., Hayward, CA, USA). Histograms were drawn using FlowJo software, version 9 for Macintosh (Tree Star, Inc., Ashland, OR).

### Assessment of plasma membrane electric potential (ΔΨ_p_)

Estimation of ΔΨ_p_ was monitored by measuring the increase in absorbance of bis-(1,3-diethylthiobarbituric acid) trimethine oxonol [DiSBAC_2_(3)] (Invitrogen) as previously described [24]. Briefly, *L. amazonensis* promastigotes (2×10^7^/mL) were added into black polystyrene 96-well microplates in HBSS+Glc containing 0.2 µM DiSBAC_2_(3) in a final volume of 100 µL per well. The plate was incubated at 25°C in a microplate reader (POLARstar Omega, BMG Labtech) and fluorescence was recorded (λ_ex_ = 544 nm; λ_em_ = 584 nm) every 2 min. After signal stabilization raloxifene was added to final concentrations of 15, 30 or 60 µM. Gramicidin D 8 µM (Sigma-Aldrich) was used as a positive control. Untreated parasites and parasites incubated with the highest volume of diluent (DMSO 0.6%) were used as negative controls. No interference in DiSBAC_2_(3) fluorescence was observed when raloxifene was added to HBSS+Glc in the absence of cells. Three independent experiments were performed, each one with triplicate samples.

### Estimation of mitochondrial transmembrane electric potential (ΔΨ_m_)

Changes in ΔΨ_m_ were monitored by flow cytometry, using the fluorescent dye rhodamine 123 (Rh123) (Sigma-Aldrich) as previously described [24]. *L. amazonensis* promastigotes (2×10^7^/mL) were resuspended in HBSS+Glc and incubated (1 mL final volume) with increasing concentrations of raloxifene at 25°C for 20 min. After treatment, parasites were washed twice in HBSS+Glc, loaded with Rh123 (0.3 µg/mL, 10 min, 37°C), washed twice in HBSS+Glc and analysed by flow cytometry. Fluorescence emission was quantified using the CytoSoft 4.2.1 software as described above. Parasites depolarized with 100 µM carbonyl cyanide 4-(trifluoromethoxy)phenylhydrazone (FCCP, Sigma-Aldrich) were used as a positive control. Untreated parasites and parasites incubated with the highest volume of diluent (DMSO 1.2%) were used as negative controls. Results were obtained from three independent experiments.

### Evaluation of raloxifene's activity *in vivo*

Female BALB/c mice (4 to 5 weeks-old) were inoculated intradermally with 1×10^6^ stationary-phase *L. amazonensis* promastigotes at the proximal end of the tail, as described [12]. Three weeks after infection, mice were randomly assigned into experimental groups. In a first series of experiments (n = 2), mice infected with wild type *L. amazonensis* were assigned to groups (n = 10) that were either left untreated or received 40 mg/kg/day raloxifene by oral gavage in 100 µL final volume of saline. Treated animals received 10 doses of raloxifene, given on weekdays.

A second series of experiments (n = 2) was performed with mice infected as described above, except for the use of the luciferase expressing parasites, LaLUC. Treated groups (n = 5) received 100 mg/kg/day raloxifene prepared with Cremophor A25 (Sigma-Aldrich) (100 mg/mL) by oral gavage in 100 µL final volume. The control group received 100 µL of the vehicle used to dilute raloxifene. All animals received 10 doses of the assigned scheme, given on alternate days.

In both series of experiments, disease progression was evaluated once a week by recording the average diameter of the tail measured as the mean of tail base diameters in horizontal and vertical directions. Measurements were taken with a caliper (Mitutoyo Corp., Japan). For the second series of experiments, parasite burden was evaluated at the end of the treatment (6 weeks post-infection) through luciferase detection by bio-imaging (IVIS Spectrum, Caliper Life Sciences, Inc. MA/USA) as described [17]. Briefly, prior to imaging, mice received 75 mg/kg luciferin (VivoGlo Luciferin, Promega) intraperitoneally. Imaging was collected 20 min later, through high-resolution mode from a fixed-size region of interest. Results were quantified with Living Image software version 4.3.1 (Caliper Life Sciences), and results were expressed as ph/sec/cm^2^/sr. Animal experiments were approved by the Ethics Committee for Animal Experimentation (Protocol 033/42/02).

### Statistical analysis

*In vitro* data were analysed for statistical significance by One-way ANOVA, followed by the Tukey post-test. Data on lesion progression and parasite burden were analysed for statistical significance by using the non-parametric Mann-Whitney test. Statistical analyses were performed using GraphPad Prism 5 software.

## Results

### *In vitro* activity of raloxifene against *Leishmania* spp. and mammalian cytotoxicity

The activity of raloxifene was tested initially against promastigotes of different *Leishmania* species incubated in culture medium supplemented with FCS. Sensitivity was uniform across the genus, with EC_50_ values ranging from 30.2 to 38.0 µM after 24 h incubation (Table 1).

**Table 1:** Half maximal effective concentration (EC_50_) of raloxifene against *Leishmania* spp.

*Leishmania* species	Stage^a^	Condition (medium or buffer)	Time of incubation (hours)	EC_50_ ± SD^b^
*L. amazonensis*	P	M199	24	30.2±1.7
*L. amazonensis*	P	M199	2	30.6±1.0
*L. amazonensis*	P	HBSS+Glc	2	9.3±1.0
*L. braziliensis*	P	M199	24	38.0±8.4
*L. donovani*	P	M199	24	32.5±1.3
*L. infantum chagasi*	P	M199	24	30.9±0.5
*L. major*	P	M199	24	32.6±1.4
*L. mexicana*	P	M199	24	30.3±1.3
*L. amazonensis*	LA	RPMI	24	15.0±2.3
*L. amazonensis*	IA	RPMI	48	16.2±2.2
*L. infantum chagasi*	IA	RPMI	48	8.8±1.1

a Parasite form: P: promastigotes; LA: lesion derived amastigotes; IA: intracelullar amastigotes. Half maximal effective concentration against promastigotes and axenic lesion-derived amastigotes was determined through MTT or MTS as described [20]; activity against *L. amazonensis* and *L. infantum chagasi* intracellular amastigotes was determined through luciferase activity or microscopic counting, respectively, as described in Material and Methods.

b The results are expressed as the mean and standard deviation (± SD) of three independent experiments, each one performed in triplicate.

Maximal effect was already observed after 2 h incubation with raloxifene in M199 medium, with an EC_50_ value of 30.6±1.0 µM against *L. amazonensis* promastigotes. The antileishmanial activity was more pronounced when the assay was carried out in HBSS+Glc, with an EC_50_ of 9.3±1.0 µM after 2 h incubation (Table 1).

Cytotoxicity against the host cells *in vitro* was determined using cultures of J774 macrophages treated with raloxifene for 24 h with calculated CC_50_ of 28.6±0.5 µM.

Drug activity was also tested *ex vivo* against *L. amazonensis* amastigotes obtained from mice infected tissue allowing the determination of an EC_50_ of 15.0±2.3 µM. Similar activity was demonstrated when raloxifene was used to treat cultures of BMDM infected with *L. amazonensis* or *L. infantum chagasi* (Table 1, Figure S1). Raloxifene's Selectivity Index varied between 1.76 and 3.24, for *L. amazonensis* and *L. infantum chagasi* intracellular amastigotes, respectively.

The EC_50_ of miltefosine, a control standard drug against *L. amazonensis* promastigotes and intracellular amastigotes was calculated as 16.8±1.7 µM and 2.7±0.3 µM, respectively, in agreement with previously published data [25].

### Ultrastructural alterations caused by raloxifene in *L. amazonensis* promastigotes and intracellular amastigotes

**Figure 1:**
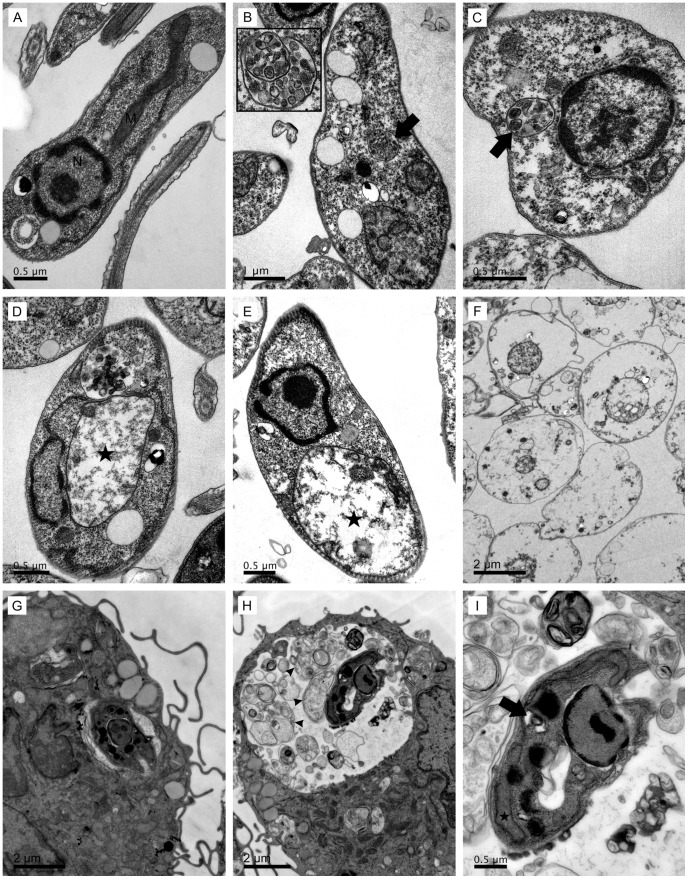
Ultrastructural aspects in raloxifene treated *Leishmania* promastigotes and intracellular amastigotes. (A to F): Ultrathin sections of *L. amazonensis* promastigotes. Promastigotes untreated (A) or incubated with 60 µM raloxifene for 30 min (B and C), 2 h (D and E) or 14 h (F) in M199 observed under transmission electron microscopy. The structure indicated by the arrow in (B) is shown in higher magnification in the inset. (G to I): Ultrastructural morphology of intracellular amastigotes. BMDM were infected with *L. amazonensis* and cultured for 48 hours in the absence (G) or in the presence of 9 µM raloxifene (H and I). (I) shows the same field as in (H) under higher magnification. Raloxifene induced the formation of autophagosomes (arrows) and mitochondrial swelling (stars), with no disruption of the plasma membrane. Arrowheads indicate remnants of vacuolated cell bodies compatible with dead amastigotes. N: nucleus; M: mitochondrion.

Transmission electron microscopy was used to investigate the effects of raloxifene on the parasite's ultrastructure and morphology. Untreated promastigotes showed typical ultrastructure (Figure 1A) while parasites treated with raloxifene displayed morphological alterations as early as 30 min after incubation with the drug, when vacuoles similar to autophagosomes were observed (Figure 1B and C, arrows). After 2 h, severe mitochondrial damage was noted with marked swelling and loss of the matrix content (Figure 1D and E, stars). In all cases the plasma membrane seemed to be continuous. The absence of membrane disruption was observed even in promastigotes treated with raloxifene for 14 h, which presented severe damage of the cytoplasm (Figure 1F).

In infected macrophages treated with raloxifene for 48 hours, parasitophorous vacuoles were filled with remnants of vacuolated cell bodies compatible with dead amastigotes (Figure 1H, arrowheads). The ultrastructure of intracellular amastigotes in infected BMDM also displayed morphological alterations compatible with the formation of autophagosomes (Figure 1I, arrow) and mitochondrial swelling (Figure 1I, star).

### Cells treated with raloxifene display autophagic vacuoles

**Figure 2:**
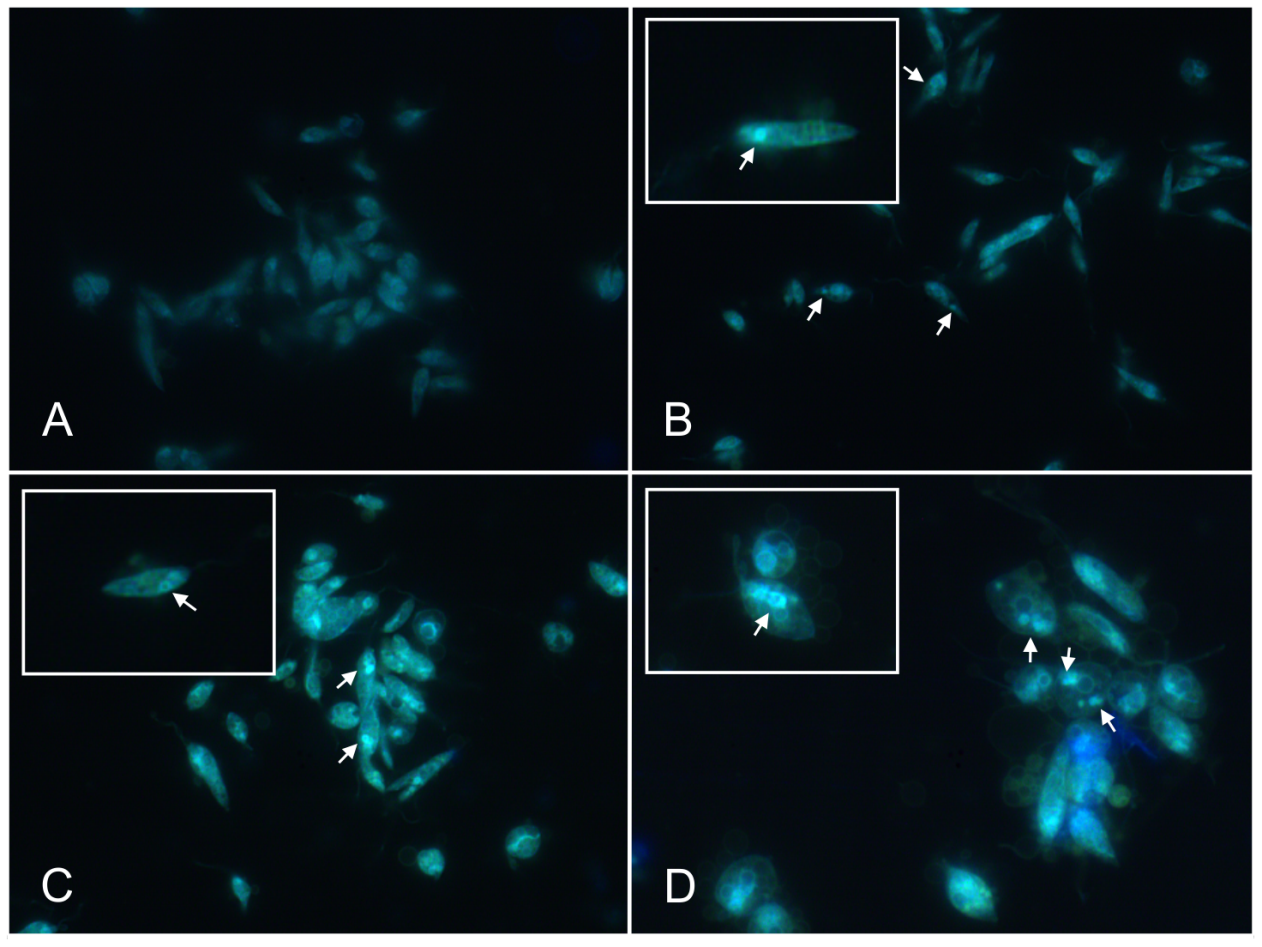
Monodansylcadaverine (MDC)-labeling of raloxifene-treated promastigotes. *L. amazonensis* promastigotes were incubated at 25°C in M199 medium (A), in PBS for 3 h (B), with 30 µM (C) or 60 µM raloxifene (D) in M199 medium for 30 min. Following the incubation period, cells were labeled with MDC, observed by fluorescence microscopy and photographed under 630×magnification (A to C) or 1,000×magnification (D). Prominent cytoplasmic MDC-labeled vacuoles are indicated by arrows. Images are representative of two independent experiments. The insets show increased size images of cells displaying MDC-positive vacuoles.

MDC is considered a marker for the presence of autophagosomes [26]. Raloxifene-treated promastigotes were labeled with MDC and visualized by fluorescence microscopy. Spherical structures stained by the dye were observed in parasites treated with raloxifene for 30 min (Figure 2C and D), in contrast to the weak and diffuse overall cytoplasmic staining in untreated parasites (Figure 2A). PBS starvation was used as a known inducer of autophagy [27]. In these conditions promastigotes concentrated the label in round bodies (Figure 2B) with a pattern similar to the one observed after treatment with raloxifene.

### Raloxifene does not disrupt *L. amazonensis* membrane integrity

**Figure 3:**
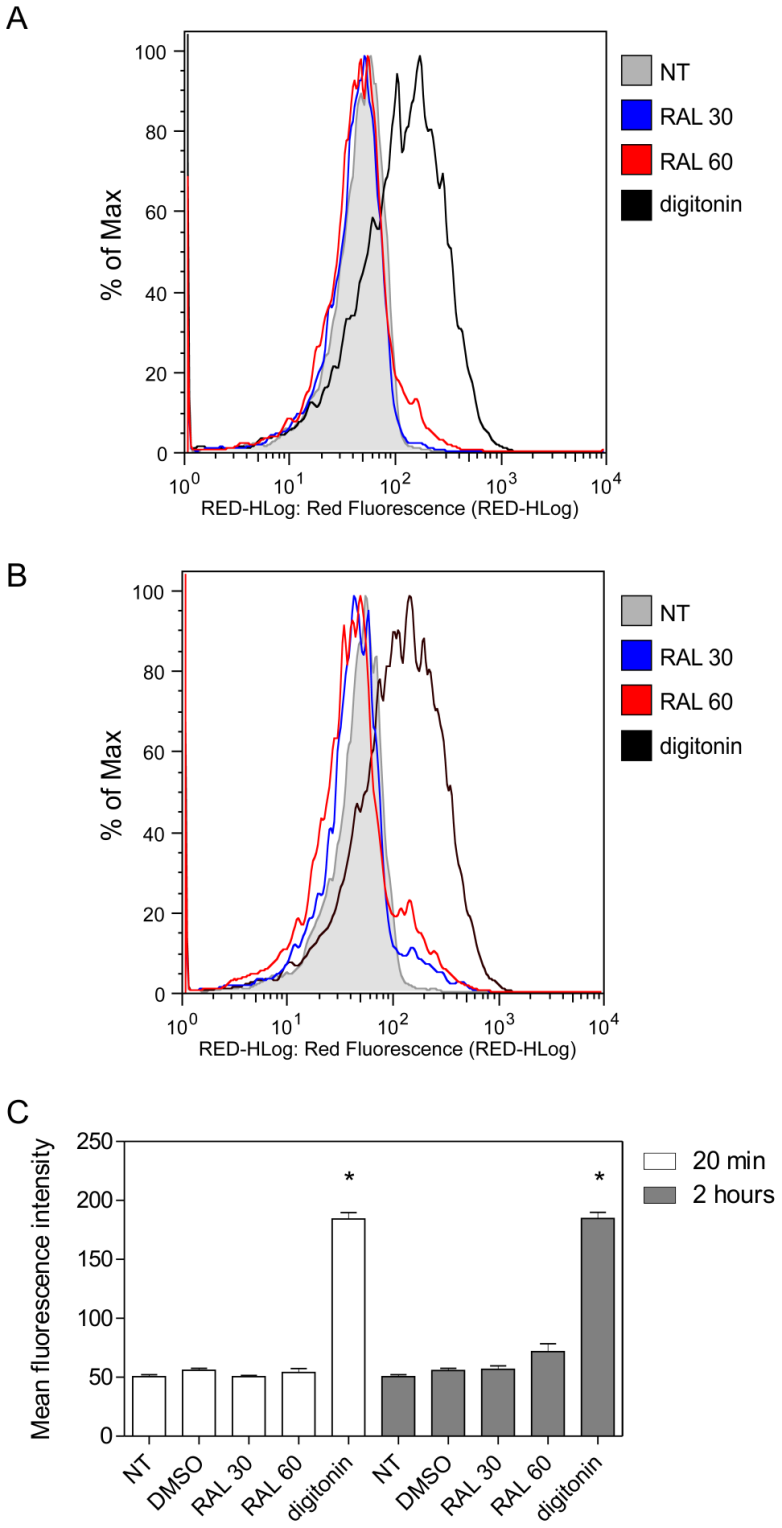
Propidium iodide labeling after raloxifene treatment. Parasites were incubated at 25°C in M199 medium, left untreated or treated with 30 or 60 µM raloxifene and analysed upon addition of propidium iodide. Parasites treated with 25 µM digitonin were used as a positive control. Untreated parasites and parasites incubated with the highest volume of drug diluent (DMSO 0.6%) were used as negative controls. Fluorescence histograms are representative of three independent experiments with untreated parasites (gray), 30 µM raloxifene (blue), 60 µM raloxifene (red) or 25 µM digitonin (black) for 20 min (A) or 2 hours (B). (C) Values shown are the mean fluorescence intensity ± standard deviation of three independent experiments. (*) indicates significant difference relative to the untreated group (p<0.0001). NT: untreated; RAL: raloxifene.

Membrane integrity can also be evaluated through permeation of vital dyes such as PI. Permeation of PI in promastigotes treated with raloxifene for 20 min or 2 h was measured by flow cytometry. While parasites treated with 25 µM digitonin showed early and pronounced increase in the uptake of PI, indicative of membrane damage, treatment with 30 or 60 µM raloxifene did not induce any significant changes in fluorescence after 20 min or 2 h (Figure 3).

### Raloxifene alters the ΔΨ_p_

**Figure 4:**
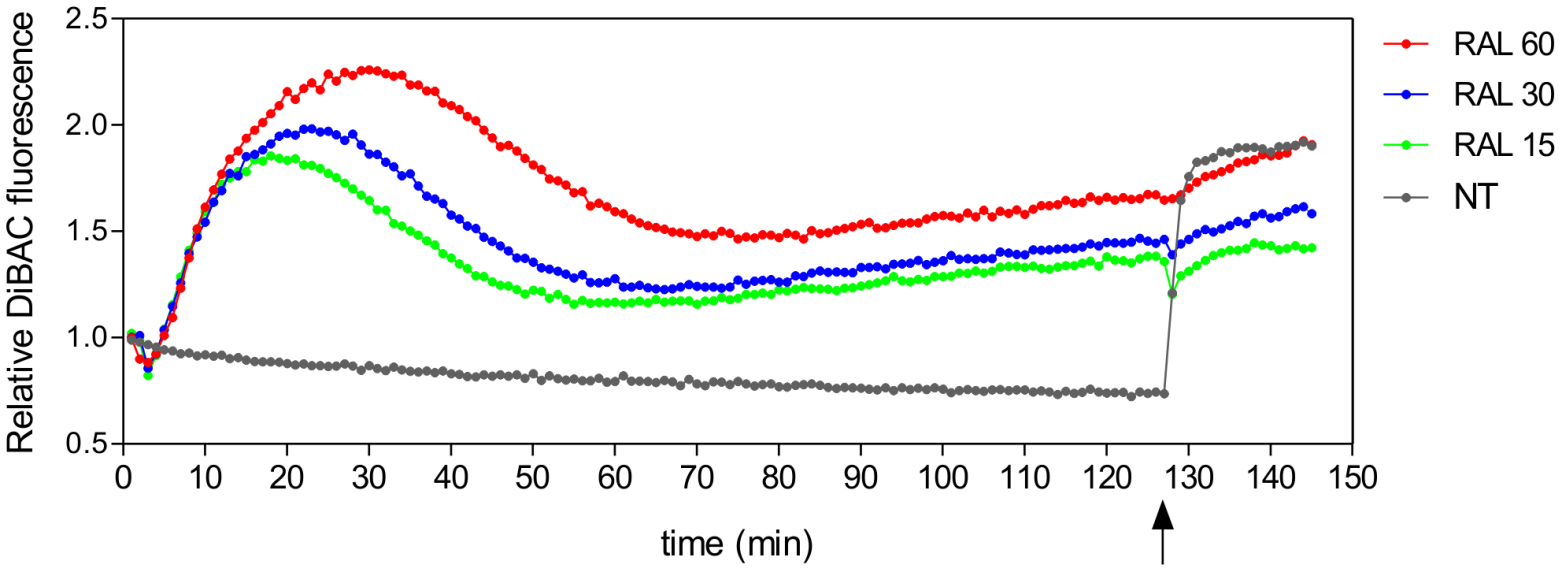
Plasma membrane potential in raloxifene treated parasites. *L. amazonensis* promastigotes were equilibrated with 0.2 µM DiSBAC_2_(3) in Hank's balanced salt solution supplemented with glucose at 25°C. Fluorescence was recorded continuously (544 nm excitation, 584 nm emission) and drugs were added at time 0. Cells were treated with 15, 30 or 60 µM raloxifene. At the end of the experiment (indicated by the arrow), 8 µM gramicidin was added to all wells. Traces are from one experiment representative of three independent experiments.

To verify whether raloxifene alters plasma membrane functions which could not be observed through transmission electron microscopy or PI staining, ΔΨ_p_ was monitored using DiSBAC_2_(3) as a fluorescent probe. In cells loaded with the voltage sensitive fluorescent dye, raloxifene induced an early decrease in fluorescence indicating membrane hyperpolarization (Figure 4). This early effect was followed, after the first 5 min of treatment, by a dose-related fluorescence intensity increase to reach levels indicative of total depolarization after 30 min incubation. This was confirmed by treatment with the nonselective ionophore gramicidin, which only marginally increased the fluorescence in cells previously treated with raloxifene while a significant increase in fluorescence was noted in control untreated parasites.

### Raloxifene induces loss of ΔΨ_m_

**Figure 5:**
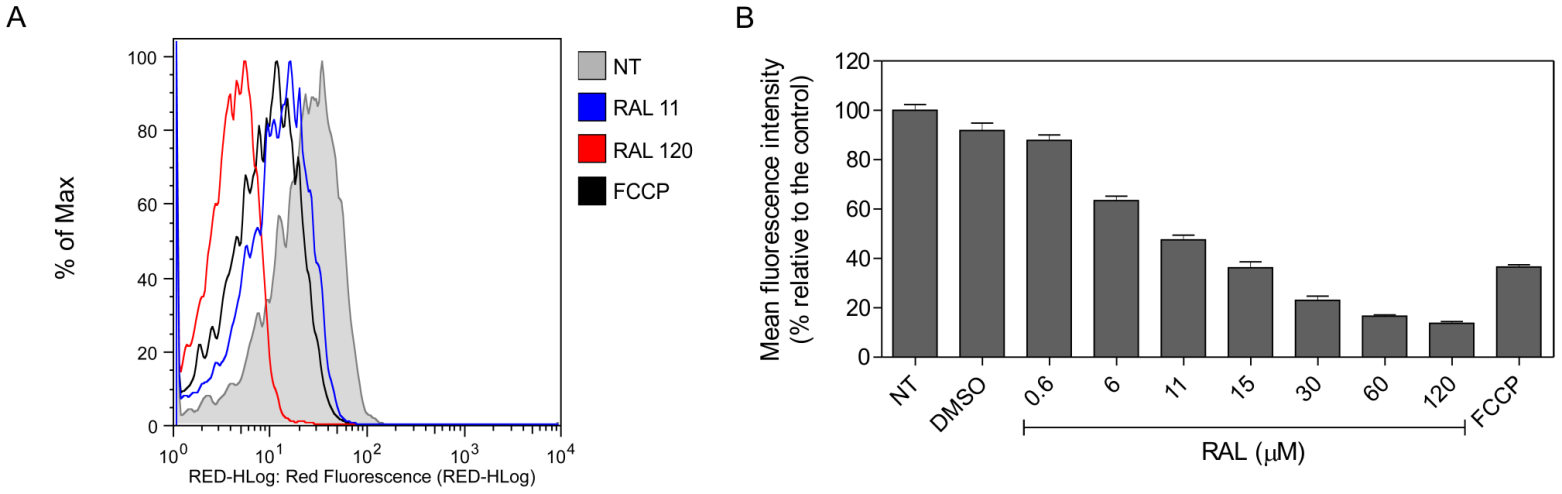
Mitochondrial membrane potential in raloxifene treated parasites. *L. amazonensis* promastigotes preincubated with raloxifene in Hank's balanced salt solution supplemented with glucose for 20 min were loaded with 0.3 µg/mL Rh123, and the fluorescence level was measured by flow cytometry. Parasites treated with 100 µM FCCP were used as a positive control. Untreated parasites (NT) and parasites incubated with the highest volume of drug diluent (DMSO 1.2%) were used as negative controls. (A) Representative fluorescence histograms with untreated parasites (gray), 11 µM raloxifene (blue), 120 µM raloxifene (red) or 100 µM FCCP (black). (B) Mean fluorescence intensity compared to the control in parasites treated with increasing concentrations of raloxifene. Bars represent the mean and standard deviation of triplicates in an experiment representative of three independent experiments.

In order to confirm the mitochondrial damage observed under transmission electron microscopy, the mitochondrial function was evaluated using the fluorescent probe Rh123 by flow cytometry. Parasites treated with increasing concentrations of raloxifene for 20 min exhibited a gradual inability to concentrate the dye, indicating a progressive collapse of the ΔΨ_m_ (Figure 5). The EC_50_ calculated based on Rh123 fluorescence was 9.42±1.03 µM, in accordance with the EC_50_ based on mitochondrial activity measured in parasites incubated in HBSS+Glc for 2 h. The mitochondrial protonophore FCCP was used as positive control of mitochondrial membrane depolarization and induced a reduction of 63.5±0.9% in Rh123 accumulation.

### *In vivo* efficacy of raloxifene

**Figure 6:**
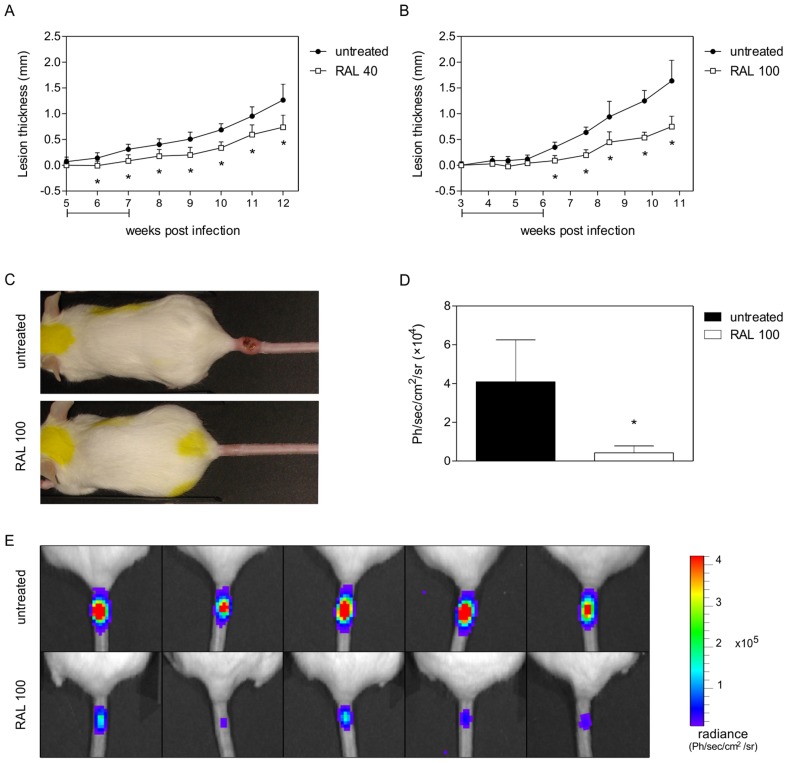
Clinical follow up and determination of parasite burden in raloxifene-treated mice. BALB/c mice were infected with 1×10^6^ promastigotes at the proximal end of the tail. Treatment was initiated 3 weeks post-infection. (A) Mice infected with wild type *L. amazonensis* and treated with 40 mg/kg/day raloxifene for 10 doses in weekdays (n = 10/group). (B–E) Mice infected with LaLUC and treated with 100 mg/kg/day raloxifene in a total of 10 doses in alternate days (n = 5 per group). (A and B) Progression of lesion thickness (mean ± standard deviation) in untreated (circles) and raloxifene-treated mice (squares). The horizontal bar indicates the period of raloxifene administration. (C) Macroscopical aspect of the lesion in representative animals from the untreated and treated groups. (D) Lesion bioluminescence (mean ± standard deviation) recorded from untreated and raloxifene-treated mice 6 weeks post-infection. (E) Bioluminescence imaging from untreated and raloxifene-treated mice 6 weeks post-infection. Ph/sec/cm^2^/sr: photons per second per square centimeter per steradian. (*) *p*<0.01 in comparison with untreated group. Results shown are from one experiment representative of two independent experiments.

Treatment of *L. amazonensis*-infected BALB/c mice was initiated 3 weeks post-infection and the progression of lesion thickness in untreated and raloxifene-treated mice was recorded weekly. In a first series of experiments, treated mice received 40 mg/kg/day raloxifene orally for 10 doses on weekdays. Significant reduction in lesion thickness in treated groups was noticed (Figure 6A). Five weeks after the end of treatment, the average size of lesions in treated groups was reduced by 41.7% as compared with control mice. After the interruption of treatment, lesion size in treated groups increased but did not reach sizes observed in control untreated animals (Figure 6A).

In a second series of experiments, mice were treated with 100 mg/kg/day orally in a total of 10 doses in alternate days. In this case, significant decrease in the lesion size was more pronounced with a 54.3% reduction in the average lesion size of treated mice five weeks after the end of treatment (Figure 6B and C). In order to estimate the parasite burden in the site of infection, light emission of LaLUC parasites was recorded at the end of raloxifene treatment (6 weeks post-infection). Parasite burden was reduced by 89.7% in the group treated with raloxifene when compared with the untreated group, as indicated by bioluminescence quantification (Figure 6D and E). Untreated and vehicle-treated groups were not significantly different (data not shown).

## Discussion

The activity of the triphenylethilene tamoxifen against *Leishmania*, *in vitro* and *in vivo* was previously demonstrated [12], [14], [15]. Tamoxifen and raloxifene are classified as SERMs, a class of compounds that exhibit agonist and antagonist estrogenic effects depending on the target tissue. Here, we report that raloxifene – a benzothiophene belonging to a SERM class distinct from tamoxifen's – also presents *in vitro* broad-spectrum activity against different *Leishmania* species. This is the first report of the activity of raloxifene against protozoan parasites.

Tamoxifen has been shown to be strongly incorporated into biomembranes, to disrupt membrane structure and to induce mitochondrial permeabilization [28], characteristics that can be correlated with its high hydrophobicity (LogP = 5.93) and are estrogen receptor-independent. Raloxifene is also highly lipophilic (LogP = 5.69) and possesses estrogen receptor independent activities [29]. The observation of continuous plasma membrane ultrastructure as well as the lack of increased permeability to PI in raloxifene-treated promastigotes indicated absence of membrane disruption. On the other hand, more subtle membrane lesions can be monitored using potential-sensitive fluorescent probes such as DiSBAC_2_(3) which enters depolarized cells where binding to intracellular proteins causes enhanced fluorescence. Interestingly, treatment with raloxifene induced an early hyperpolarization followed by dissipation of the membrane potential. The early event may be due to fast ionic currents, observed as drug interaction with the membrane takes place. This is followed by the collapse of ionic gradients across the membrane leading to depolarization. At this point, it is unclear whether the loss of membrane potential is due to a disarrayed membrane structure or to inhibition of ion channels.

Interestingly, previous studies have shown that raloxifene inhibits voltage dependent calcium currents in mouse spermatogenic cells in an estrogen receptor independent way [30]. The presence of a voltage gated calcium channel sharing several characteristics with the human counterpart has been recently demonstrated in the plasma membrane of *Leishmania* [31]. Accordingly, calcium channel blockers used as anti-hypertensive drugs, have been shown to possess antileishmanial activity [32], [33], [34]. Thus, interference in calcium channels in raloxifene treated parasites cannot be ruled out.

Raloxifene was previously shown to decrease ΔΨ_m_ in human endometrial carcinoma cells [35]. In raloxifene-treated parasites we observed severe mitochondrial damage by transmission electron microscopy and Rh123 labeling. As a result, the energy-coupling system in the mitochondria is most likely inactivated.

The observation of autophagic vacuoles by transmission electron microscopy and the presence of MDC-labeling vacuoles in raloxifene-treated promastigotes suggest the participation of autophagy in raloxifene's mode of action. This is in accordance with previous findings indicating that SERMs induce autophagy in tumor cells [36], [37]. Autophagosomes and MDC-labeled vacuoles have been previously described as effects of antileishmanial compounds [38], [39].

Previous to raloxifene-induced cell death, loss of mitochondrial membrane potential and entrapment of cytoplasm content within autophagosomes were noted. The presence of autophagic vacuoles is most likely related to the degradation of damaged organelles induced by raloxifene treatment. Based on the obtained results, we propose the following sequence of events triggered by raloxifene: i) partition of raloxifene in the plasma membrane; ii) alteration in plasma membrane potential; iii) mitochondrial membrane depolarization; iv) mitochondrial dysfunction; v) autophagy; vi) parasite death.

Raloxifene was developed as an anti-estrogen against breast cancer and was later found out to be a therapeutic agent for postmenopausal osteoporosis. Pharmacokinetic studies have shown good oral absorption; glucuronide conjugates are formed after extensive first-pass metabolism in the intestinal mucosa and liver. Acute toxicity is low with no mortality observed with doses up to 5000 mg/kg orally in mice and rats. Preclinical toxicology studies indicated that raloxifene was well tolerated in repeated dose assays in mice, rats, dogs and monkeys. In mice, daily doses of up to 120 mg/kg raloxifene for 3 months induced no serious toxic effects [40]. Based on this data, we chose the starting oral dose of 40 mg/kg/day (3 times the human dose for osteoporosis treatment and prevention, based on body surface area) [41] for initial *in vivo* tests in a murine model of cutaneous leishmaniasis. Treatment of *L. amazonensis* infected BALB/c mice with raloxifene resulted in significant decrease in lesion size. As the lesion in treated animals did not heal, we then increased the dose to 100 mg/kg/day. As the drug is poorly soluble in water, for this second series of experiment raloxifene was prepared in Cremophor A25, which is a vehicle capable of yielding stable emulsions of hydrophobic, pharmacologically active biomolecules.

Treatment of *L. amazonensis* infected BALB/c mice with 100 mg/kg/day raloxifene resulted in significant decrease in lesion size and parasite burden. No toxic effects were observed during drug administration. Raloxifene-treated animals did not heal the lesions completely and, after the interruption of treatment, lesions worsened. However, these lesions did not reach sizes observed in untreated controls. Since BALB/c mice infected with *L. amazonensis* represents a model of extreme susceptibility, the significant reduction in the number of parasites in treated animals supports the proposal of further testing of this drug in other animal models of leishmaniasis. Furthermore, these data warrant the consideration of this molecule as a lead for further development. In fact, recent results from our laboratory indicate that the antileishmanial potency of synthetic benzothiophenes is increased 10-fold (as compared to raloxifene) by the presence of two basic side chains in the molecule [42]. Furthermore, structure-activity data showed that the most active antileishmanial benzothiophenes lack the pharmacophore for estrogen receptor activity confirming that the antileishmanial activity observed for benzothiophenes is independent of the interaction with the estrogen receptors [42].

In conclusion, the results of this work extend the investigation of SERMs as potential candidates for leishmaniasis treatment. Raloxifene's activity *in vitro* is mediated by functional damage to the plasma and mitochondrial membranes, which culminate in cell death. Further studies are necessary to ascertain whether other antileishmanial mechanisms are engaged *in vivo*.

## Supporting Information

Figure S1 Activity of raloxifene against intracellular *L. infantum chagasi* amastigotes. BMDM were infected with stationary phase promastigotes of *L. infantum chagasi* for 3 h. After washing the remaining extracellular parasites, raloxifene was added to the culture media. After 48 h incubation, slides were fixed and stained. (A) Control untreated cells; (B) 3.75 µM; (C) 5.62 µM; (D) 7.5 µM; (E) 11.25 µ; (F) 15 µM raloxifene. Bar = 10 µM.
(TIF)
