# Supplementary figures and images for: Antileishmanial Activity of the Estrogen Receptor Modulator Raloxifene
Source: PLoS Negl Trop Dis. 2014 May 8;8(5):e2842. doi: 10.1371/journal.pntd.0002842 (PMC4014391; doi:10.1371/journal.pntd.0002842)

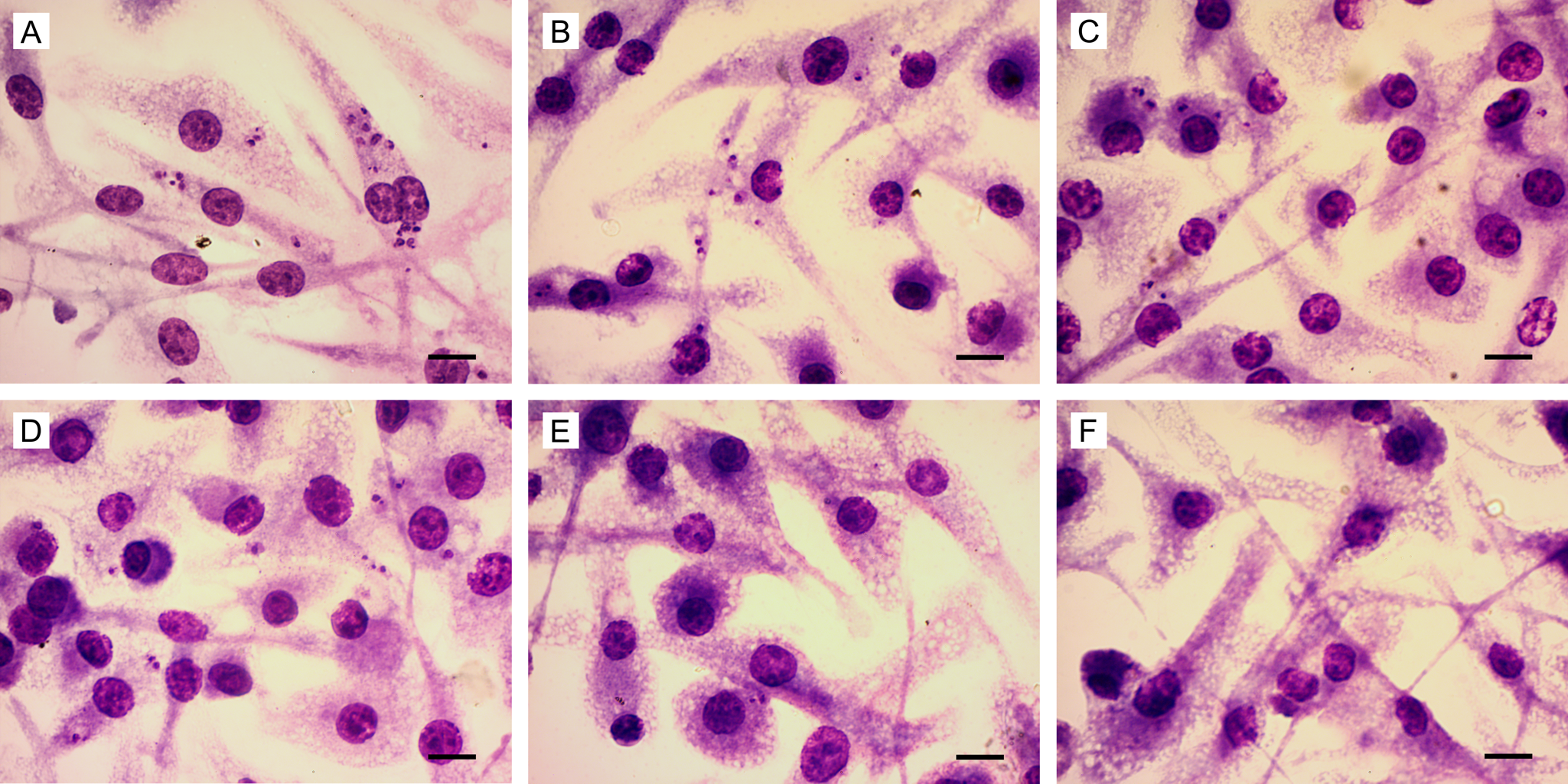

Supplement: Figure S1 — Activity of raloxifene against intracellular L. infantum chagasi amastigotes. BMDM were infected with stationary phase promastigotes of L. infantum chagasi for 3 h. After washing the remaining extracellular parasites, raloxifene was added to the culture media. After 48 h incubation, slides were fixed and stained. (A) Control untreated cells; (B) 3.75 µM; (C) 5.62 µM; (D) 7.5 µM; (E) 11.25 µ; (F) 15 µM raloxifene. Bar = 10 µM. (TIF) [file pntd.0002842.s001.tif]
